# Narrative Co-Evolution in Hybrid Social Networks: A Longitudinal Computational Analysis of Confucius Institutes

**DOI:** 10.3390/e27121240

**Published:** 2025-12-08

**Authors:** Ming Huang, Jun-Ling Wang, Zi-Ke Zhang

**Affiliations:** 1 International Communication Institute, Zhejiang University, Hangzhou 310058, China; huangming_bellah@zju.edu.cn (M.H.); zkz@zju.edu.cn (Z.-K.Z.); 2 College of Media and International Culture, Zhejiang University, Hangzhou 310058, China; 3 School of Physics, Zhejiang University, Hangzhou 310027, China; 4 Hangzhou International Innovation Institute, Beihang University, Hangzhou 311115, China

**Keywords:** hybrid social networks, confucius institutes, complex discourse dynamics, cross-platform analysis

## Abstract

This study investigates the complex dynamics of public discourse surrounding Confucius Institutes (CIs) across the hybrid social networks of mainstream news and social platforms from 2010 to 2023. Employing a longitudinal, multi-platform design, we analyzed news articles and tweets using a computational framework combining topic modeling and sentiment analysis. Our results reveal a shared cross-platform narrative evolution from a “culture-first” to a “politics-central” orientation. However, the trajectory differed significantly: mainstream media underwent a gradual, policy-oriented shift, while social media exhibited an abrupt, nonlinear transition. Crucially, we identify an asymmetric interdependence: Twitter sentiment reliably Granger-causes mainstream media sentiment, establishing its role as a leading indicator, and systematic asymmetries in thematic framing reflect the divergent logics of each platform. The study demonstrates that public discourse on contested, state-linked institutions operates as a complex adaptive system, where bottom-up affective reactions and top-down editorial processes continuously interact in a dynamic equilibrium, ultimately co-constructing a fragmented public understanding.

## 1. Introduction

The rise of hybrid social networks has fundamentally transformed the circulation of information and the formation of collective narratives, giving rise to a communication ecology marked by inherent complexity. In this environment, mainstream media and social platforms operate as interdependent yet heterogeneous subsystems, where editorial logics, algorithmic curation, and user-driven communication converge to shape public discourse. As a result, the communication landscape can no longer be understood through isolated or hierarchical models but must be analyzed as a complex adaptive system—one in which multiple scales of interaction, from micro-level engagement to macro-level agenda evolution, continuously co-evolve.

Within this broader transformation, the global discourse surrounding Confucius Institutes (CIs) offers a paradigmatic case of communicative complexity. Established as Chinese government-supported language and culture promotion centers, CIs have become contested symbols in global communication—variously framed as instruments of cultural exchange, soft-power diplomacy, or political influence. This multiplicity of meanings generates rich discursive interactions across media platforms, reflecting underlying tensions between institutional narratives and decentralized public interpretations. Moreover, episodic policy shocks—such as the closure of CIs in the United States and European parliamentary resolutions—provide natural experimental conditions for observing how external shocks perturb the media ecosystem.

However, few studies have systematically examined how CI narratives co-evolve across mainstream and social media over extended periods. Most prior research isolates these subsystems, overlooking the adaptive, non-hierarchical, and path-dependent nature of information diffusion in hybrid environments. To capture this communicative complexity, a longitudinal and computational framework is required—one capable of quantifying both the structural and dynamic properties of the evolving discourse.

To address this gap, our study employs a multi-modal computational framework to analyze the CI discourse from 2010 to 2023 across mainstream news and Twitter. We integrate topic models with a bidirectional LSTM for sentiment analysis, enabling a system-level examination of narrative dynamics. This research aims to (1) characterize and compare the structural evolution of topic communities and sentiment in both media sectors; (2) uncover dynamic, cross-platform interdependencies, including lead–lag relationships in sentiment and thematic salience; and (3) identify systematic asymmetries in how mainstream and social media co-frame specific topics—thereby revealing the mechanisms through which complexity manifests in the hybrid social network of a contested public issue.

## 2. Related Works

### 2.1. Complexity and Social Network Analysis

Social networks are characterized by heterogeneous structures, nonlinear dynamics, and continuously evolving interactions among individuals and communities. Research in complex network theory has been instrumental in decoding the underlying mechanisms of information diffusion, opinion formation, and collective behavior in digital societies [1,2]. Foundational work on network topology [3] and comprehensive reviews of information spreading [2] provide the analytical backbone for this approach. Key to this understanding are concepts such as community, which reveals how homophilic groups form and solidify, often leading to phenomena like echo chambers [4,5]; influence propagation, which models how ideas and behaviors spread through networks; and system dynamics, which captures the temporal evolution of these networks under internal and external pressures. The rise of digital platforms has added layers of complexity, as algorithms now play a critical role in mediating visibility, structuring user interactions, and potentially fostering polarization [6]. Understanding these algorithmic ecosystems is paramount, as they govern the flow of information and shape the very architecture of public discourse [7]. The rise of digital platforms has introduced additional complexity, as algorithms mediate visibility, structure interactions, and potentially foster polarization [6]. Understanding these algorithmic ecosystems is essential, as they govern information flow and shape public discourse [7]. While existing work offers valuable theoretical and methodological insights, most studies focus either on network topology, diffusion mechanisms, or platform-specific behaviors in isolation. Few investigations integrate multi-platform data with joint modeling of topic–opinion coevolution over extended timeframes.

### 2.2. Information Diffusion in Hybrid Media Systems

The contemporary media landscape is best understood as a hybrid media system [8], where legacy outlets and social platforms are deeply intertwined, co-constructing public agendas through dynamic, often reciprocal, processes. This environment enables a wider range of actors to influence political and social discourse, transforming information diffusion into a complex, non-hierarchical phenomenon. While traditional agenda-setting theory posits that mass media influence public perceptions of issue salience [9], the hybrid system reveals a more complex picture. Empirical studies have shown that the relative influence of each sector is not fixed but varies by context and issue. For instance, while newspapers may lead online forums on some topics [10], Twitter discussions can precede or even drive traditional coverage during breaking events [11]. Recent research leveraging platforms such as Reddit further illustrates how platform-specific features shape interaction dynamics and emotional expression [12]. Despite these advances, existing studies are often constrained by short time windows, single-event analyses, or limited modeling of cross-platform sentiment dynamics. Moreover, many intermedia studies treat traditional and social media as bounded systems rather than components of a co-evolving information environment. There remains a pressing need for longitudinal, multi-platform analyses that capture both the structural and affective complexity of information diffusion in hybrid media systems.

### 2.3. Confucius Institute Narratives as a Case for Intermedia Analysis

Since their inception in 2004, Confucius Institutes (CIs) have operated at the intersection of cultural diplomacy and soft-power projection. While officially framed as language- and culture-teaching centres, CIs have frequently been portrayed in Western media as instruments of Chinese state influence or propaganda. Research on Confucius Institutes (CIs) as instruments of soft power has yielded mixed findings. Some studies report that CIs enhance China’s image and foster local partnerships—e.g., in the United States and Thailand [13] and across regions with CI presence [14]. By contrast, analyses using global surveys and public diplomacy frameworks find no clear improvement in diplomatic standing attributable to CIs [15,16,17], and in certain contexts local actors express skepticism toward CI activities [18]. Research from the host-country audience perspective remains limited, though evidence suggests general ambivalence [13] and conditional increases in favorability when CIs deliver visible community benefits or emphasize cultural-language programming over political themes [19].

As liminal cultural entities—simultaneously educational resources and diplomatic emissaries—CIs offer a compelling case for examining how contested narratives evolve across mainstream and social media. Their ambiguous status (as “Eastern other,” soft-power tool, or security threat) generates a dense network of associated concepts (e.g., “academic freedom,” “propaganda,” “cultural exchange”), making them ideal for analyzing cross-platform topic communities and sentiment dynamics. Moreover, given episodic policy shifts (e.g., U.S. closures beginning in 2014, European parliamentary resolutions in 2021), the CI case lends itself to longitudinal analysis within hybrid media systems. Existing CI research typically adopts policy analysis or examines single-platform media coverage, leaving unanswered questions regarding how CI-related narratives and sentiments co-evolve across heterogeneous media environments over time. This study uses the CI case to examine cross-platform topic communities, sentiment trajectories, and the systemic asymmetries that shape their evolution within a hybrid media system.

In summary, while existing research provides valuable insights into network dynamics, hybrid media systems, and Confucius Institute perceptions, several drawbacks remain. First, studies on complex networks often focus on single platforms or isolated mechanisms, with limited integration of multi-platform data and topic-opinion coevolution. Second, analyses of hybrid media systems typically examine short timeframes or single events, offering incomplete views of cross-platform dynamics. Third, research on Confucius Institutes has relied mainly on policy analysis or single-platform coverage, leaving their cross-platform narrative evolution underexplored. These limitations point to the value of a longitudinal, multi-platform approach that can track topic communities, sentiment trajectories, and systemic asymmetries. This study seeks to address these aspects through the case of Confucius Institutes, aiming to contribute a more integrated perspective on discourse dynamics in hybrid media environments.

## 3. Methodology

### 3.1. Data Collection

The study utilized two complementary corpora to capture the evolving discourse and sentiment surrounding the Confucius Institute across mainstream and social media platforms.

**Mainstream Media:** Google News data were collected from 1 January 2010 to 1 October 2023, yielding 5229 articles and over 5 million words related to “Confucius Institute”. Google News aggregates reports from diverse, globally distributed outlets, ensuring high coverage and representativeness. All publicly accessible sources—including state-affiliated and independent media—were retained, as they collectively constitute the information environment shaping audience perceptions. The dataset includes article text, publication timestamps, headlines, and URLs, forming the *Confucius Institute News Corpus*.

**Social Media:** Twitter data from 1 January 2010 to 1 March 2023, were collected using the hashtag #ConfuciusInstitute and the keyword “Confucius Institute”. The final dataset comprised 16,000 tweets (≈380,000 words), forming the *Confucius Institute Twitter Corpus*. Given Twitter’s role as a dynamic and high-velocity communication platform, these data provide valuable insights into real-time user interactions and opinion diffusion. Standard preprocessing (cleaning, language filtering, tokenization, and lemmatization) ensured analytical consistency across corpora.

Both datasets were transformed into standardized text formats suitable for topic and sentiment analysis, thereby enabling cross-platform comparison of temporal trends and narrative alignment.

### 3.2. Research Approach

**Figure 1:**
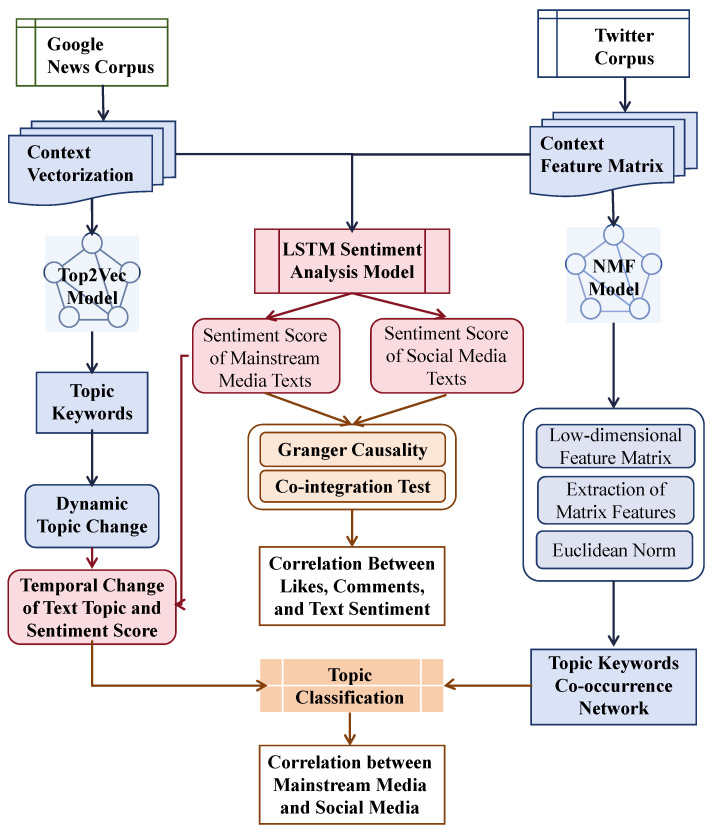
The data processing framework.

This study implemented a data-driven natural language processing (NLP) framework (Figure 1) that integrates topic community, sentiment analysis, and cross-platform trend mapping to reveal the dynamic information flow within hybrid media system.

Two complementary analytical streams were constructed:

1. **Topic community:** For long and structured texts (mainstream news), the *Top2Vec* algorithm was employed to discover latent semantic communities. For short, sparse texts (tweets), *Non-negative Matrix Factorization (NMF)* was used as a text-clustering technique to extract coherent themes from high-dimensional data.
2. **Sentiment Analysis:** Both corpora were evaluated using a unified bidirectional LSTM sentiment model, ensuring comparability across media environments. The resulting sentiment series enable the exploration of temporal co-fluctuations and cross-platform diffusion patterns.

Time-indexed sentiment trajectories were subsequently fitted with polynomial functions, and cosine similarity between topic centroids across time windows was calculated to trace inter-platform narrative convergence.

#### 3.2.1. Top2Vec for Mainstream News

Mainstream news articles, being long and editorially curated, are well-suited for embedding-based semantic clustering. The *Top2Vec* model jointly learns document and topic embeddings through Doc2Vec and applies density-based clustering (HDBSCAN) to automatically identify coherent topic groups [20]. This neural embedding architecture allows for the detection of semantically close documents and dynamic topic formation without predefining topic numbers.

In this work, the model was implemented with the following key parameters: Doc2Vec embeddings were trained using speed=“learn” with eight worker threads. UMAP dimensionality reduction was performed with its default configuration (*n*_neighbors_ = 15, *n*_components_ = 5, min_dist = 0, cosine metric), and HDBSCAN clustering was applied using standard settings (min_cluster_size = 15, min_samples = 15). Under these specifications, Top2Vec automatically inferred the final number of topics based on the density structure of the embedding space. In this study, 65 topics finally emerged organically from the embedding space.

Compared to traditional probabilistic models (e.g., LDA), Top2Vec (1) captures fine-grained semantic nuances through contextual embeddings, (2) determines topic granularity adaptively, and (3) efficiently scales to large corpora—advantages validated in prior work [21]. This step enables the mapping of long-term narrative evolution and structural shifts in mainstream discourse networks.

#### 3.2.2. NMF for Twitter Data

Twitter texts are short, sparse, and often noisy. Non-negative Matrix Factorization (NMF) has been found to perform well on short texts such as tweets [22], and comparative evaluations report that NMF (together with embedding-based approaches like BERTopic) often yields clear, human-interpretable topic separations on Twitter data. While BERTopic can produce novel insights through its embedding workflow, NMF typically provides relatively “standard” but stable and readily interpretable topic factors—an attractive property for downstream network and diffusion analyses [23]. NMF also does not require strong prior assumptions about topic structure [24], scales well to large short-text collections, and has demonstrated robustness to noise in social-media contexts [25].

After extracting topics using NMF, the study employed k-means clustering to further calculate the associations within and between topics. A random term (TF-IDF vector) from each topic was selected as a cluster centroid, denoted as $T_{c}$. For each topic vector $T_{i}$ in the dataset, the Euclidean distance was calculated as

$$d_{k}=∥T_{c}-T_{i}∥.$$

These centroids $T_{c}$ were then treated as nodes, with the distances $d_{ic}$ as edges, to construct a co-occurrence graph.

#### 3.2.3. LSTM-Based Sentiment Analysis

To quantify emotional dynamics, a pretrained bidirectional LSTM model was employed using the “Sentiment140” dataset [26]. This model classifies text polarity (positive, neutral, negative) and provides a standardized sentiment scoring scheme applicable across both corpora. The dataset was divided into training (80%) and test (20%) subsets, with the tokenizer vocabulary limited to 10,000 tokens and sequence length set to 200.

The model architecture included:

- an embedding layer (60 dimensions) transforming token indices into dense representations;
- a bidirectional LSTM layer (64 neurons) capturing contextual dependencies;
- a dropout layer (rate 0.5) mitigating overfitting; and
- a dense output layer with sigmoid activation producing sentiment probabilities.

**Figure 2:**
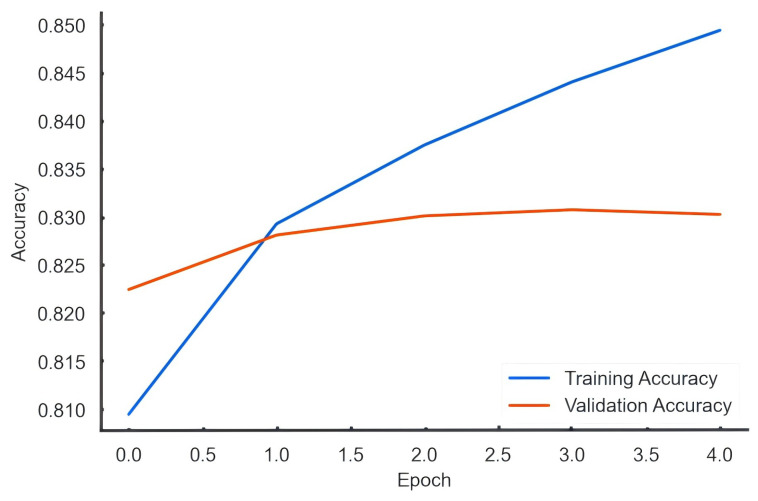
Accuracy and Validation Accuracy Curves over Epochs.

Trained with the Adam optimizer and binary crossentropy loss, the model achieved an accuracy of approximately 83% (Figure 2). Sentiment scores were normalized to the range $\left[-1,\;1\right]$ and aggregated at the document level to generate temporal sentiment trajectories. These trajectories enable the detection of emotional fluctuations and diffusion patterns across hybrid media environments.

## 4. Results

### 4.1. Narrative Discourses and Their Evolution Across Platforms

This section examines the topic communities and temporal evolution of the Confucius Institute (CI) discourse, comparing the distinct narrative ecosystems of mainstream media and social media.

#### 4.1.1. Topic Community and Evolution in Mainstream Media

Top2Vec automatically produced 68 distinct topic centroids for the mainstream-news corpus. To extract CI-related narratives, each centroid was compared (cosine similarity) with a manually created “Confucius Institute” seed vector; the ten highest-scoring topic centroids were retained for detailed analysis (Appendix A lists the top 30 keywords for each retained topic).

**Figure 3:**
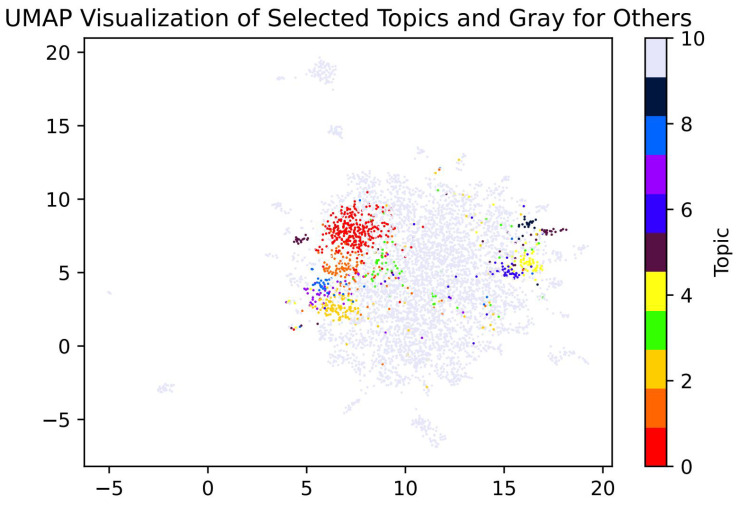
UMAP visualization of Top2Vec-derived document embeddings. Dense local clusters coexist with more diffuse bridging nodes (e.g., Topic 34), indicating overlapping semantic communities in mainstream coverage.

The UMAP projection in Figure 3 visualizes the semantic landscape of these topics. It shows several tightly clustered topics alongside a more diffuse cluster (Topic 34, in green), indicative of a pervasive narrative thread that does not coalesce into a single, focused discourse. This diffuse topic primarily represents the framing of CIs as language-teaching and exam-preparation centers. Keywords related to standardized tests like the HSK and China’s Gaokao system appear across a wide range of articles—from promotional pieces to broader discussions on language proficiency. This diffuseness underscores the Institutes’ embedded role in the global language-education ecosystem, a theme that permeates the CI narrative rather than being confined to a specific debate.

**Figure 4:**
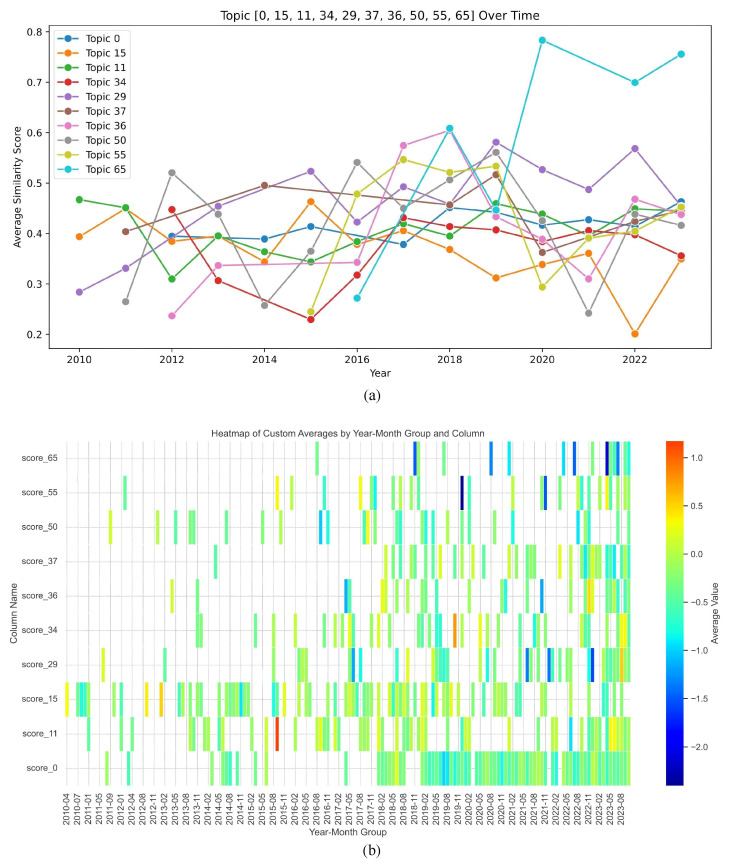
(**a**) Temporal evolution of clustered topics (annual basis); (**b**) Heatmap of sentiment scores for topics with high correspondence to CI-related discourse.

The longitudinal analysis reveals a significant diversification of mainstream coverage from 2010 to 2023 (Figure 4a). In the initial period (2010), discourse was dominated by three core topics: organizational activities (Topic 29), pedagogical methods (Topic 15), and cultural festivals (Topic 11). These themes, covering events like the Spring Festival and the “Chinese Bridge” competition, were generally framed in neutral tones, establishing CIs primarily as cultural and educational entities.

The period 2011–2012 saw an increase in thematic diversity, though still anchored in educational quality and language instruction. Sentiment remained predominantly neutral (Figure 4b), with occasional fluctuations reflecting debates over academic standards. The COVID-19 pandemic acted as a external shock, causing a sharp decline in topics related to offline activities (e.g., Topic 11 and Topic 50) in 2020–2021, with only a partial recovery in 2022. Topic 15 (teaching modes) notably declined, likely a consequence of pandemic-related disruptions to teacher dispatches.

Across the entire period, a politically oriented topic community (Topic 0, encompassing funding transparency, academic governance, and party links) remains consistently present as an undercurrent and exhibits negative-tone spikes around 2019–2020. In sum, mainstream discourse manifests stable thematic modularity with episodic reconfiguration under exogenous shocks, a pattern consistent with gradual evolution in mainstream media.

#### 4.1.2. Topic Community Trajectory on Social Media

**Figure 5:**
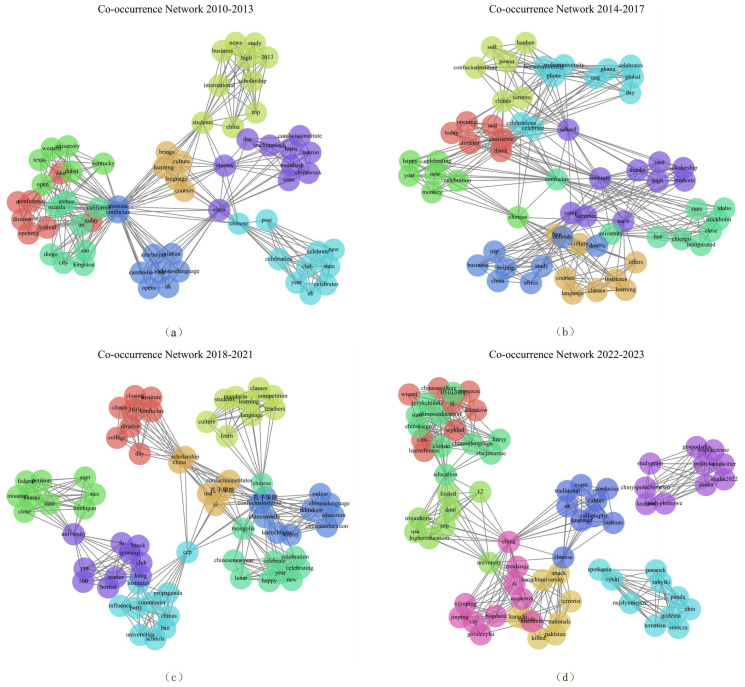
Co-occurrence graphs (3-year windows) derived from clustered Twitter topics: (**a**) 2010–2013; (**b**) 2014–2017; (**c**) 2018–2021; (**d**) 2022–2023. Node colors indicate topic membership; edge density indicates keyword co-occurrence strength.

Twitter data were analyzed with NMF as a text-clustering tool and aggregated in multi-year windows (2010–2013, 2014–2017, 2018–2021, 2022–2023) to construct time-sliced topic co-occurrence networks (see Figure 5) (nodes = keywords; edges = co-appearance within tweets).

2010–2013: The discourse network was characterized by tight, well-defined clusters around pedagogical and cultural themes such as “learning,” “students,” and “festivals” (Figure 5a). This community reflects an initial, largely apolitical public interest in the Institutes’ core educational mission.

2014–2017: The topic community began a marked shift (Figure 5b). While cultural exchange remained present, new clusters emerged around keywords like “propaganda,” “influence,” and “state,” signaling the integration of geopolitical skepticism into the discourse and foreshadowing a major narrative transition.

2018–2021: A profound structural reconfiguration occurred, with political frames becoming dominant (Figure 5c). Clusters around “closure,” “policy,” and “USA” grew central, dwarfing earlier cultural themes. This reflects the period where CIs became potent symbols within broader security and soft-power anxieties.

2022–2023: The network solidified around a core of political critique (Figure 5d). Dense interconnections around “censorship,” “transparency,” and “funding” highlight sustained public engagement with issues of governance and trust.

In summary, social media discourse underwent a non-linear transition from a decentralized, culturally-oriented network to centralized and politicized communities. These shifts indicate (i) increasing semantic centralization of political frames on Twitter over time and (ii) a progressive change in modular structure from multiple loosely connected cultural clusters to fewer, denser political modules.

In sum, a comparative analysis reveals a shared macro-level trajectory—from “culture-first” to “politics-central”—but also critical differences in pace and framing logic. Mainstream media exhibited a gradual, policy-oriented shift, intensifying political scrutiny around major geopolitical events. Social media, in contrast, displayed an abrupt transition, rapidly entwining and then foregrounding political frames by 2014–2017. Both platforms responded coherently to external shocks like the COVID-19 pandemic, de-emphasizing offline activities. However, each retained vestiges of its original narrative focus, indicating path dependence in their respective discourse ecosystems.

### 4.2. System-Level Dynamics: Influence and Co-Evolution

This section investigates the dynamic interactions and mutual influences between the two platforms, characterizing the hybrid media system.

#### 4.2.1. Sentiment as a Leading Indicator and Long-Term Coupling

**Figure 6:**
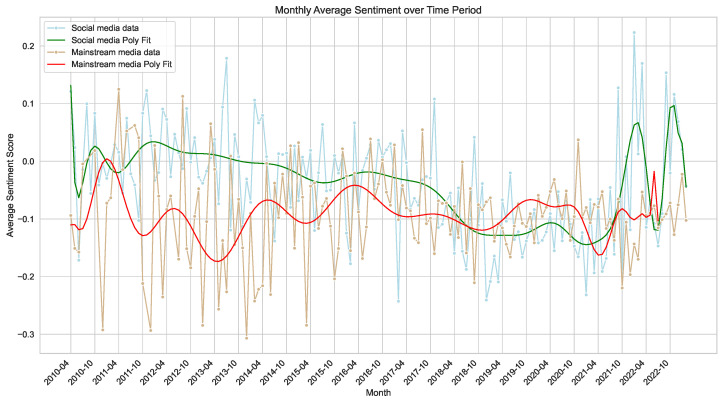
Comparison of Monthly Average Sentiment Overlap Fitting Curves (2018–2023).

Because reliable Twitter data were only available from 2018 onward, the analysis period was restricted to 2018–2023 to ensure temporal consistency and statistical precision. Monthly sentiment averages for both mainstream and social media were used to construct polynomial trend curves (Figure 6). Visual inspection indicated possible phase shifts between the two trajectories, suggesting a lead–lag coupling between sentiment dynamics across platforms.

Prior to testing directional relationships, the Augmented Dickey–Fuller (ADF) test confirmed that both sentiment indices were stationary, thereby meeting the precondition for causal inference. The results (Twitter: ADF = −3.118, p=0.025; Mainstream: ADF = −4.014, p=0.001) establish a stable foundation for further time-series analysis.

Subsequent Granger causality testing revealed a unidirectional influence from Twitter sentiment toward mainstream sentiment, while the reverse path was not statistically significant (Table 1).

To evaluate long-term interdependence, the Johansen cointegration test was applied. Both the trace and maximum eigenvalue statistics reject the null hypothesis of no cointegration, confirming a stable long-term equilibrium between the two sentiment indices (Table 2).

These findings delineate a two-scale coupling mechanism: short-term sentiment perturbations on social media precede corresponding movements in mainstream sentiment, yet both series co-evolve toward a shared long-term equilibrium. This configuration typifies a hierarchical coupling process in hybrid information networks—where high-frequency, user-driven fluctuations propagate upward and gradually synchronize with slower, institutionally mediated responses.

**Figure 7:**
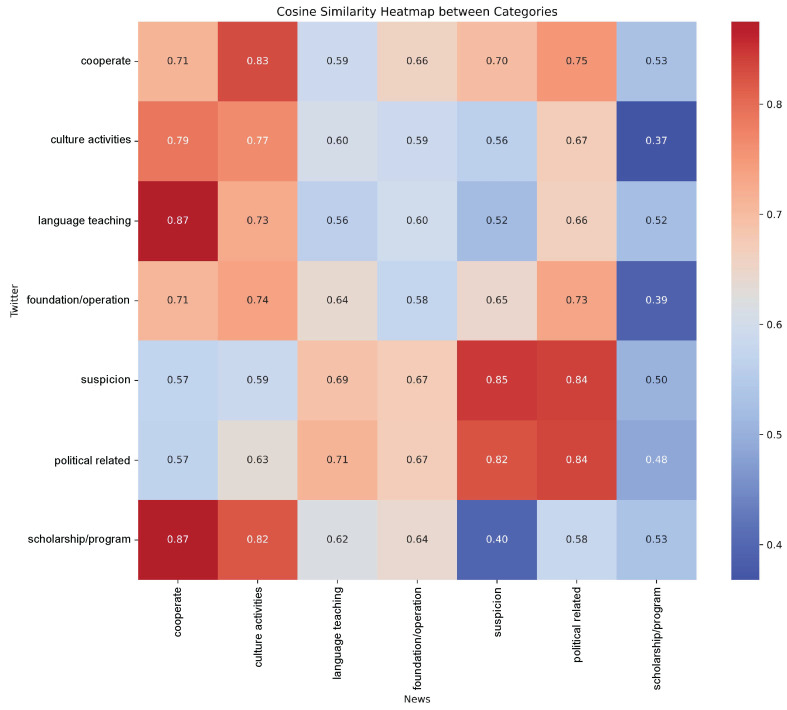
Topic Similarity Matrix between Mainstream Media and Social Media.

The phase-shifted peaks observed in Figure 6 coincide with identifiable geopolitical and policy events, including the COVID-19 pandemic (2020) and subsequent discourse on “Suspicion” and “Political” frames (Figure 7). Negative sentiment surges typically originate on Twitter, where distributed users rapidly mobilize around clusters of keywords such as “closure,” “propaganda,” and “funding.” These clusters then diffuse into mainstream discourse through recontextualization in news coverage, reflecting a bottom-up contagion of emotional intensity. Conversely, when legacy outlets introduce new institutional narratives—such as policy statements or the establishment of new CI branches—the resulting social media responses exhibit more heterogeneous sentiment, ranging from endorsement to skepticism.

This asymmetric interaction indicates that social media functions as an early-phase excitatory subsystem within the broader information diffusion, generating rapid emotional perturbations that precede editorial adaptation. Over extended time horizons, however, the two systems display convergence in their sentiment baselines, evidencing a macro-level synchronization driven by iterative feedback between public emotion and journalistic framing. This pattern could represents a form of multi-layer coupling between fast (Twitter) and slow (mainstream) subsystems: perturbations emerge at the periphery through decentralized user discourse, diffuse through intermedia links, and stabilize as shared evaluative frames within the institutional layer. The resulting long-term coherence illustrates how collective sentiment in complex socio-technical systems evolves through intertwined short-term reactivity and long-term adaptation processes.

#### 4.2.2. Asymmetry in Thematic Networks

The topic similarity matrix (Figure 7) depicts the structural coupling of topic salience between mainstream media and Twitter, where warmer hues denote stronger co-occurrence patterns and cooler hues indicate divergence. This matrix captures not only the degree of thematic convergence but also the asymmetric dependencies that characterize the hybrid information ecosystem surrounding CIs.

Distinct clusters of high and low similarity reveal heterogeneous coupling intensities across thematic domains. High similarity values—“Suspicion” (0.85), “Political Related” (0.84), “Culture Activities” (0.77), and “Cooperate” (0.71)—represent tightly synchronized attention. These domains exhibit temporally co-evolving information flows, suggesting that geopolitical framings and cultural representations diffuse in parallel across media layers. Particularly, the synchronized evolution of suspicion and political scrutiny indicates strong cross-platform resonance, where both systems amplify narratives framing CIs as geopolitical instruments. Meanwhile, “Culture Activities” maintain steady cross-platform coherence, reflecting a shared resonance around participatory cultural events and linguistic exchange.

In contrast, low similarity values—“Scholarship/Program” (0.53), “Language Teaching” (0.56), and “Foundation/Operation” (0.58)—indicate decoupled dynamics and differentiated narrative anchoring. On Twitter, these topics gravitate toward micro-level experiences (e.g., individual scholarships, classroom interactions), while mainstream media frames them through macro-level institutional and policy lenses. The divergence illustrates scale-specific information structuring: social discourse emerges from bottom-up, experiential aggregation, whereas mainstream narratives operate through top-down institutional codification.

Beyond mean similarity levels, the matrix uncovers systematic asymmetries in co-framing directionality. When social discourse foregrounds cooperation narratives—academic partnerships or joint programs—mainstream media exhibits a correlated increase in suspicion coverage (similarity 0.70). However, the reverse link is weaker (0.57), implying unidirectional sensitivity: institutional narratives in mainstream media more readily trigger political reinterpretations on social platforms than vice versa. A similar asymmetry manifests in the language-teaching frame. Twitter’s pedagogical focus corresponds to low mainstream-to-social coupling (0.52), whereas institutionalized educational debates in the press induce higher social amplification of oversight concerns (0.69). This directional dependency reflects a form of conditional coupling, where politically charged or policy-oriented signals in the mainstream network modulate the sentiment and attention flows in social media.

An inverted configuration appears in the Scholarship/Program cluster: cooperation narratives on Twitter attract limited mainstream response (0.53), yet mainstream reporting on institutional cooperation induces significant social amplification of scholarship-related content (0.87). This reversal delineates the non-reciprocal coupling between system scales. While mainstream media diffuses information through formalized, top-down structures, social media reorganizes it through decentralized interest-driven pathways, generating emergent resonance around personal benefit rather than policy abstraction.

Collectively, these asymmetric linkages outline a complex coupling architecture within the hybrid media system. Rather than reflecting linear agenda transmission, the observed co-framing patterns indicate multi-scale informational interactions governed by directional dependencies, temporal feedback, and selective amplification. The resulting communication field operates as a dynamic network in which institutional, cultural, and personal narratives continuously interact—producing a state of partial synchronization between structural coherence and interpretive divergence.

## 5. Discussion

### 5.1. The Co-Evolution of Narrative Ecosystems

Our findings depict the discourse around Confucius Institutes (CIs) not as a linear process, but as the co-evolution of two coupled narrative ecosystems—mainstream media and social media—each with distinct internal dynamics yet interacting within a larger information environment.

Both platforms initially converged on a “culture-first” narrative, framing CIs around pedagogical and cultural functions. This initial state represents a relatively stable, low-complexity attractor in the public discourse, consistent with the Institutes’ stated mission. However, this stability proved to be transient. Driven by external geopolitical shocks and internal platform dynamics, the narrative systems underwent a significant phase transition towards a “politics-central” state.

The trajectory and rate of this transition, however, were platform-dependent. Mainstream media exhibited a gradual, inertial shift, characterized by incremental introductions of political scrutiny. This reflects the high institutional friction and editorial gatekeeping inherent in legacy media systems. In contrast, social media underwent an abrupt and radical reconfiguration, with political frames rapidly eclipsing cultural ones. This nonlinear, rapid response is a hallmark of complex adaptive systems with low barriers to entry and high virality potential, where grassroots skepticism can quickly gain traction and reshape the entire discourse network [27]. The convergence of both systems on a politicized equilibrium, despite different paths, underscores the powerful influence of macro-level geopolitical context on seemingly decentralized information ecosystems.

### 5.2. Platform-Driven Asymmetries in Narrative Framing

The observed asymmetries in thematic framing and cross-platform influence stem from the fundamentally divergent logics of information processing that characterize each platform.

Mainstream media operates with a macro-systemic logic, consistently framing issues through lenses of policy, development, and geopolitics. In this logic, a story about institutional cooperation is inherently a story about diplomatic strategy, thereby triggering associative links to “suspicion” and state influence. Social media, conversely, is driven by a personal-utility logic, where information is evaluated based on its affective resonance and relevance to individual experience [28]. Here, cooperation is interpreted as scholarship opportunities and classroom experiences.

This divergence explains the non-reciprocal topic similarity from asymmetric similarity matrix. The strong reaction from social media to mainstream coverage of cooperation (similarity = 0.87) occurs because the press’s macro-narrative is successfully translated by users into personally relevant terms. The weaker reverse flow (similarity = 0.53) happens because the personal, experiential narratives dominant on Twitter lack the inherent geopolitical salience required to easily bypass the gatekeeping filters of legacy media. This creates a feedback loop where the press’s structural narratives are constantly being translated, resisted, or reframed through the affective and personal lenses of the social media sphere, leading to a fragmented public cognition of the CI phenomenon.

### 5.3. System Dynamics and Social Media as a Leading Indicator

The Granger causality from Twitter to mainstream media sentiment, coupled with their long-term cointegration, positions social media as a leading indicator and critical amplifier within the system. This unidirectional influence suggests that Twitter functions as a high-resolution sensor for shifts in public affect, particularly negative skepticism, often crystallizing around keywords like “closure” and ”propaganda” in response to external events. These grassroots sentiment cascades then seep into the mainstream, which acts with a delay due to its institutional inertia. The role of social media, as uncovered in this study, transcends that of a mere communication channel; it functions as a complex adaptive public sphere. Its capacity to serve as a leading indicator for mainstream sentiment and a site for asymmetric framing is deeply intertwined with its algorithmic architecture. Platforms like Twitter are engineered to prioritize engagement, which often amplifies content that is novel, affective, and controversial [27]. Our observed correlation between negative sentiment and higher comment volumes is a direct manifestation of this design, creating a built-in incentive structure for the spread of skepticism.

This environment fosters the emergence of counterpublics—subaltern spaces where dominant narratives are contested and alternative frames are generated [29,30]. In the CI context, social media users collectively constructed a narrative emphasizing personal agency and local impact, countering the macro-political frames prevalent in mainstream coverage. While this empowers marginalized voices and enriches pluralism, it also contributes to the fragmentation of public discourse. When these counterpublic discourses become siloed and amplified by engagement-driven algorithms without cross-cutting dialogue, they risk forming echo chambers that hinder consensus-building [6].

Psychological mechanisms further underpin these dynamics. The Elaboration Likelihood Model (ELM) [31,32] offers a plausible explanation for the observed divergence. The fast-paced, cue-rich environment of social media likely encourages peripheral-route processing, where attitudes are formed based on emotional associations and heuristics. In contrast, the more structured context of mainstream media may facilitate central-route processing for certain audiences, involving more deliberate consideration of arguments. These divergent processing routes can lead to the formation of differentially stable attitudes across the public. Furthermore, Attribution Theory [33] helps explain the persistent skepticism: the consensus, distinctiveness, and consistency of negative frames around CIs as state-linked entities collectively drive attributions of malign intent, reinforcing a cycle of distrust that is resilient to disconfirming evidence.

### 5.4. Limitations and Future Research

This study has several limitations that point toward future research in computational social science. First, the data collection via Google News and Twitter’s API is subject to strong algorithmic curation, which may bias the sample towards highly engaging or algorithmically promoted content. Such selection effects could amplify certain sentiment cascades or topic clusters while underrepresenting less visible narratives. Future work could integrate additional, less curated sources or explicitly model these algorithmic biases to better capture the full spectrum of discourse.

Second, the method for extracting CI-related narratives relied on a manually defined “Confucius Institute” seed vector and cosine similarity. While this approach was supplemented with cross-validation and manual inspection to ensure relevance, it may not capture all variations of discourse. Future research could explore more data-driven techniques, such as embedding-based clustering or iterative seed expansion, to improve coverage and robustness.

Third, the use of different topic models (Top2Vec and NMF), though strategically adaptive for heterogeneous data, introduces comparability challenges. Future studies could investigate unified frameworks for consistent cross-platform analysis. Moreover, the present study did not conduct systematic sensitivity checks on model parameters, which should be addressed in future work to further validate the robustness of the findings.

Finally, our sentiment analysis relied on a general-purpose model. For geopolitically charged discourse, future research would benefit from domain-specific sentiment lexica or fine-tuned models to improve accuracy. From a complexity science perspective, future work could also employ agent-based modeling to simulate the observed cross-platform dynamics or use dynamic network analysis to track the real-time flow of frames between platforms.

## 6. Conclusions

This study has leveraged a multi-modal computational framework to dissect the complex, co-evolutionary dynamics of public discourse surrounding Confucius Institutes (CIs) across the hybrid media landscape from 2010 to 2023. By moving beyond linear models of influence and embracing a complexity-oriented perspective, we have mapped the intricate interplay between narrative structures, sentiment flows, and platform-specific logics that characterize this contested socio-technical system.

The principal contributions of this study are threefold. Methodologically, it advances an integrative analytical pipeline that models topic–sentiment coevolution while accounting for algorithmic mediation. Empirically, it provides evidence that social media functions as a high-frequency leading indicator and that platform logics systematically generate asymmetric reframings of contested issues. Conceptually, it offers a complexity-oriented perspective that reconceptualizes public discourse as a coupled narrative ecosystem shaped by nonlinear dynamics and platform-specific amplification mechanisms.

Our analysis reveals that the discourse on CIs is not a monolithic entity but a multi-scale system composed of two coupled yet distinct narrative ecosystems. While both mainstream media and Twitter exhibited a macro-level trajectory from “culture-first” to “politics-central,” their evolutionary paths were markedly different. Mainstream media demonstrated a gradual, inertial shift, consistent with a system characterized by editorial gatekeeping and high institutional friction. In contrast, social media underwent an abrupt, nonlinear phase transition, a signature of a complex adaptive system where low barriers to entry and algorithmic amplification enable the rapid reorganization of narrative networks. This divergence underscores that the pace and nature of discourse evolution are emergent properties of the underlying platform architectures.

At the system level, we identified a clear hierarchical coupling. Twitter sentiment consistently functioned as a Granger-causal leading indicator for mainstream media sentiment, establishing its role as a high-frequency sensor for shifts in public affect. However, the long-term cointegration of the two sentiment indices confirms that these platforms are not decoupled; they exist in a dynamic equilibrium, where fast, decentralized perturbations from the social layer gradually synchronize with the slower, institutionalized responses of the mainstream. This pattern exemplifies a fundamental dynamic in hybrid media systems: the tension between rapid, bottom-up emergence and slower, top-down integration.

Furthermore, the observed asymmetries in thematic network—such as the non-reciprocal relationships between “cooperation” and “suspicion” frames—highlight the divergent information-processing logics at play. The mainstream media’s macro-systemic, policy-oriented lens consistently recontextualizes grassroots narratives through a geopolitical prism, while social media users refract institutional messages through a personal-utility and affective filter. This generates a fragmented public cognition of CIs, where different segments of the audience operate with fundamentally different “facts” and interpretive schemas, a direct consequence of the platform-driven polarization inherent in complex digital publics.

In conclusion, this research demonstrates that understanding modern public discourse requires a shift in perspective—from analyzing isolated media effects to modeling the emergent, system-level behaviors of a coupled information ecosystem. The case of the Confucius Institutes illustrates how state-linked cultural initiatives become entangled in the nonlinear dynamics of algorithmically mediated platforms, where sentiment cascades, asymmetric framing, and cross-platform feedback loops collectively shape diplomatic reputations in ways that transcend official communication strategies.

## Figures and Tables

**Table 1:** Granger causality test results.

Direction	Lag Order	F-Statistic	*p*-Value	Significance
Twitter → Mainstream	1	4.804	0.032	Significant causality
	2	3.547	0.036	Significant causality
Mainstream → Twitter	1	1.033	0.314	No significant causality
	2	1.577	0.216	No significant causality

**Table 2:** Johansen cointegration test results.

Statistic	Rank = 0	Rank = 1	95% Critical Value	Decision
Trace Statistic	13.094	1.397	12.321	Reject rank = 0, accept rank = 1
Max-Eigen Statistic	11.697	1.397	11.225	Reject rank = 0, accept rank = 1

## Data Availability

The data presented in this study are available on request from the corresponding author.

## Appendix A

**Table A1:** Mainstream Media Topics Keyword Lists.

Topic	Keywords
Topic 0	Confuciusins colleges campuses institutes programs lawmakers espionage entities universities scrutiny rubio funding oversight ccp federal funded communist authorization malign bipartisan transparency report beacon subcommittee congressional department disclose censorship programming pentagon
Topic 15	elementary immersion kindergarten programs montgomery troy grade instruction school schools classes instructors district classroom alabama program superintendent programming teacher teach classrooms teachers languages districts grades language class curriculum math educators
Topic 11	dragon festival event celebration lunar performances traditional celebrate celebrations lanterns spring attendees calligraphy lantern festivities lion dances zodiac rabbit celebrating luck dance costumes boat year calendar colorful feast reunion fun
Topic 34	hsk exam test proficiency exams language english courses learners education teachers gaokao tests study mandarin grades enrolled classes instruction scholarship speakers secondary examination certificates abroad teaching scholarships nationwide cis languages
Topic 29	contestants competition proficiency contest bridge finals rounds round finalists won language final secondary talent judges singing audience participated competitors compete competitions winners winner organized preliminary culture represent hunan speeches themed
Topic 37	namibia tcm acupuncture medicine lebanon lebanese xinhua treatments learners enrolled enthusiasm traditional healthcare learn clinic understanding healing zhang culture techniques aug chinese friendship doctors learning enabling language medical medicines youths
Topic 36	kenyan kenya nairobi kenyans language ugandan xinhua uon uganda interactions learn colleagues proficiency bachelor studying makerere fostering african bridge job sino fluent oldest chinese africa boys interact train gala perform
Topic 50	pupils bangor primary wales secondary learning teaching schools school classes learn languages mandarin teachers mep language calligraphy lessons teach curriculum learners tutors programme teacher uk grades youngsters english classrooms taught
Topic 55	wushu kung fu martial rwanda practicing tai popularity learn sword chi calligraphy teaches culture fan movies gala dances zeng henan tanzanian moves trained attracted federation performances fun arts practitioners learning tanzanian salaam tanzania dar emmanuel es secondary symposium twelve language
Topic 65	textbooks improve learn mozambique vocational urged skills talents organized learners xinhua proficiency commended envoy young opportunities celebration showcase sports representatives
